# Opportunities and Challenges When Using the Electronic Health Record for Practice-Integrated Patient-Facing Interventions: The e-Assist Colon Health Randomized Trial

**DOI:** 10.1177/0272989X221104094

**Published:** 2022-06-28

**Authors:** Jennifer Elston Lafata, Deirdre A. Shires, Yongyun Shin, Susan Flocke, Kenneth Resnicow, Morgan Johnson, Ellen Nixon, Xinxin Sun, Sarah Hawley

**Affiliations:** UNC Eshelman School of Pharmacy and UNC Lineberger Comprehensive Cancer Center, University of North Carolina at Chapel Hill, Chapel Hill, NC, USA; Center for Health Policy and Services Research, Henry Ford Health System, Detroit, MI, USA; School of Social Work, Michigan State University, East Lansing, MI, USA; School of Medicine, Virginia Commonwealth University, Richmond, VA, USA; School of Medicine, Oregon Health and Science University; School of Public Health, University of Michigan, Ann Arbor, MI, USA; UNC Eshelman School of Pharmacy, University of North Carolina, at Chapel Hill, Chapel Hill, NC, USA; Center for Health Policy and Services Research, Henry Ford Health System, Detroit, MI, USA; School of Medicine, Virginia Commonwealth University, Richmond, VA, USA; School of Medicine, University of Michigan, Ann Arbor, MI, USA

**Keywords:** colorectal cancer screening, hierarchical generalized linear models, patient portals, program reach

## Abstract

**Background:**

Even after a physician recommendation, many people remain unscreened for colorectal cancer (CRC). The proliferation of electronic health records (EHRs) and tethered online portals may afford new opportunities to embed patient-facing interventions within clinic workflows and engage patients following a physician recommendation for care. We evaluated the effectiveness of a patient-facing intervention designed to complement physician office-based recommendations for CRC screening.

**Design:**

Using a 2-arm pragmatic, randomized clinical trial, we evaluated the intervention’s effect on CRC screening use as documented in the EHR (primary outcome) and the extent to which the intervention reached the target population. Trial participants were insured, aged 50 to 75 y, with a physician recommendation for CRC screening. Typical EHR functionalities, including patient registries, health maintenance flags, best practice alerts, and secure messaging, were used to support research-related activities and deliver the intervention to enrolled patients.

**Results:**

A total of 1,825 adults consented to trial participation, of whom 78% completed a baseline survey and were exposed to the intervention. Most trial participants (>80%) indicated an intent to be screened on the baseline survey, and 65% were screened at follow-up, with no significant differences by study arm. One-third of eligible patients were sent a secure message. Among those, more than three-quarters accessed study material.

**Conclusions:**

By leveraging common EHR functionalities, we integrated a patient-facing intervention within clinic workflows. Despite practice integration, the intervention did not improve screening use, likely in part due to portal-based interventions not reaching those for whom the intervention may be most effective.

**Implications:**

Embedding patient-facing interventions within the EHR enabled practice integration but may minimize program effectiveness by missing important segments of the patient population.

**Highlights:**

- Electronic health record tools can be used to facilitate practice-embedded pragmatic trial and patient-facing intervention processes, including patient identification, study arm allocation, and intervention delivery.
- The online portal-embedded intervention did not improve colorectal cancer (CRC) screening uptake following a physician recommendation, likely in part because portal users tend to be already highly engaged with healthcare.
- Relying on patient portals alone for CRC screening interventions may not alter screening use and could exacerbate well-known care disparities.

## Introduction

Despite multiple effective screening tests, colorectal cancer (CRC) screening remains underutilized relative to other cancer-screening tests.^1^ Our research has shown that a driving factor behind this underutilization among insured individuals is the gap that exists between physician recommendation and patient receipt of care. While the overwhelming majority (93%) of patients who are due for CRC screening receive a physician recommendation during routine primary care office visits, only 54% of patients are screened in the following year.^2^ The gap in adherence to CRC screening following a physician recommendation is likely fueled by multiple factors, including suboptimal patient-provider communication during screening recommendations and inadequate logistical support in completing screening once patients leave the physicians’ office.^3–17^

How to support patients in obtaining evidence-based screening (or other services) once they have a physician recommendation remains a challenge that has implications not only for patient well-being but also for the organizations responsible for delivering their care. Using individual health navigators holds promise, especially for low-literacy patients, but costs associated with such “high-touch” interventions limit scalability.^18,19^ Numerous prior studies have found that stand-alone patient decision aids result in improvements in patient screening knowledge, risk perceptions, and related outcomes but also limited (if any) changes in screening behaviors^20–24^ and impracticalities for practice integration.^25–28^ Similarly, while patient reminders and the removal of structural barriers can increase screening use, such interventions remain disconnected from existing clinic processes^18,29–40^ and often are not sustainable outside a research environment. The effectiveness and impact of previously tested CRC screening interventions may be limited, in part, by such practice integration challenges. The proliferation of electronic health records (EHRs) and accompanying patient portals may afford new opportunities to economically engage and support patients at the time of a CRC screening or other physician recommendation for care in ways that connect with existing clinic processes yet extend beyond the boundaries of traditional office visits.

Using the patient portal functionality now commonly available within EHRs, we developed an EHR-embedded patient-facing intervention, e-Assist colon health, to assist in obtaining care following a physician recommendation for CRC screening. To ensure the intervention’s fit with practice, we partnered with clinicians and other health system staff during the design and implementation of the intervention.^41^ We hypothesized that people who received the e-Assist intervention would be more likely to complete CRC screening (primary outcome) relative to those who received links to health education documents on CRC and CRC screening (enhanced usual care). In this article, we report findings from the randomized trial used to evaluate the intervention’s effectiveness and implementation.

## Methods

### Study Design and Trial Eligibility

For the primary outcome, we evaluated the intervention’s effect on CRC screening using a 2-arm practice-embedded pragmatic trial. The trial was conducted within a large integrated health system servicing Detroit, Michigan, and the surrounding tricounty area. Trial participants included insured patients aged 50 to 75 y who were eligible for CRC screening (as recommended by the US Preventive Services Task Force at the time of study recruitment^42^). As previously reported,^43^ the trial targeted adults of average risk for CRC and thus excluded those with a personal or family history of CRC, colon polyps, inflammatory bowel disease, familial adenomatous polyposis, or hereditary nonpolyposis as well as those for whom an accelerated screening schedule had been documented in their medical record. Because the intervention was designed to address common gaps in patient-provider conversations at the time of CRC screening recommendation, trial eligibility was also limited to those with a primary care office visit that included a physician recommendation for CRC screening (as indicated by an open access colonoscopy referral or stool test order). As described below, because the intervention was embedded within the EHR, trial enrollment was also limited to patients with an activated portal account. All aspects of the trial were approved by the health system’s Institutional Review Board.

### Identifying Trial Eligible Patients

Two common EHR tools, the patient registry function and health maintenance flags, were used to identify and allocate study-eligible patients to the appropriate study arm. For study purposes, the EHR registry function was used to identify patients who, should they have a primary care visit with a recommendation for CRC screening, would meet the study inclusion criteria. As such, when a primary care physician electronically signed the notes, or “closed” an office visit encounter within the EHR, if that visit was with a patient previously identified as study eligible (i.e., the patient was included within the EHR registry) and included a stool test order or a colonoscopy referral (i.e., physician recommendation for CRC screening), the patient became study eligible.

An additional EHR tool, a health maintenance flag, was used to assist with study randomization. Because it was not technically feasible to randomize patients within the EHR in real time as they became study eligible, as we have described elsewhere,^43^ we randomized all patients included in the EHR registry, storing their allocation as a health maintenance flag that was not visible to clinicians or others accessing the EHR for patient care. This hidden flag was used to electronically allocate the appropriate intervention or enhanced usual care material to patients once they became study eligible via a primary care physician electronically signing an office visit encounter that included a CRC screening recommendation.

### Delivering Study Material

When a patient became study eligible, a series of programming rules automated the sending of study recruitment material and either the intervention or enhanced usual care material. This was achieved using the best practice advisory/alert (BPA) system within the EHR. While BPAs are generally used to send automated notifications or reminders to alert physicians and other clinical staff to important patient needs, they can be made silent or not appear as a pop-up message or task when clinical staff are using the EHR. Instead, they can operate in the background, routing notifications to specific user-owned in-boxes that can be checked at the user’s discretion.

For study purposes, when a physician electronically signed (i.e., closed) an office-visit note that contained a CRC screening order with a study-eligible patient, BPA programming logic led to a secure inbox message being sent to that patient’s online portal account. While the content of the secure message inviting the patient to participate in the trial was identical regardless of randomization allocation, the programming logic behind the BPA used information on the patient’s randomization allocation (as stored in the study’s health maintenance flag) to send patients in the 2 trial arms a different message attachment. The attachment was an online questionnaire that contained the informed consent material, a 6-item pretrial survey, and either the e-Assist intervention or links to the enhanced usual care material. The informed consent and pretrial survey material embedded within the attached questionnaire were identical regardless of study arm. The content of the remainder of the attached questionnaire differed by study arm (i.e., either the e-Assist intervention content or links to enhanced usual care material) and could be accessed only upon study consent.

### Intervention Design and Content

As described in detail elsewhere,^43^ we used the health belief model^44^ to guide the overall intervention design, including the use of a physician recommendation for CRC screening as the cue for engagement with the intervention, and self-determination theory to guide the overall tone of written messages to ensure they were autonomy supporting.^45^ People allocated to the intervention received information specifically designed to complement typical physician office-based recommendations for CRC screening. As such, e-Assist content filled 3 informational gaps that patients endorse as important yet frequently missing from patient-physician office visit discussions of CRC^3–17^: 1) information central to informed and shared decision making (i.e., information on when CRC screening is recommended, the different screening modalities and associated benefits/risks, and comparing available screening tests), 2) addressing barriers to screening, and 3) an understanding of the logistics of testing and assistance completing testing. The intervention content, all of which we were able to embed within the questionnaire function within the EHR, included text, pictures, and links to short videos. All users were presented with information reiterating that their doctor had recommended CRC screening and the benefits of CRC. User responses to questions facilitated progression through intervention content and the branching logic that determined what content was visible. The latter enabled users to select and filter what information was viewed and thus enabled the user to tailor content based on relevance and interest. For example, to ensure message salience, embedded questions inquired about the user’s readiness to be screened.^46^ Those indicating they were ready to be screened were provided with tips for completing their preferred screening test, while those indicating they were not ready to be screened were offered suggestions for how to overcome common barriers to screening. Similarly, users who indicated they were undecided about how to screen were provided with information regarding the pros and cons of different test options. Additional questions further determined the user’s preferences for additional information and thus determined the level of detail regarding those options that users saw.

Any trial participant who did not indicate they had completed CRC screening at the end of the questionnaire received a follow-up module. The content of the follow-up module was tailored to information collected during the initial module regarding screening test preferences and readiness to screen. Content was designed to use messaging consistent with a motivational counseling approach,^47,48^ to facilitate a CRC screening decision among undecided users, and to assist in test completion among those expressing a readiness to screen. Most follow-up modules were sent 2 wk after the initial module (questionnaire) was submitted. The trial’s protocol paper provides additional details regarding the intervention’s design and content.^43^

People allocated to enhanced usual care also received an EHR questionnaire. The content within that questionnaire was identical to that received by those in the intervention group in terms of consent material, pretrial survey items, and introductory material reiterating that CRC screening had been recommended for them and outlining the benefits of screening, but instead of the text, pictures, and short videos, it contained links to 4 webpages that were stored within the health system’s patient portal’s health information library. That material, distributed by Healthwise at the time of the study,^49^ contained educational information on the etiology, symptoms, and treatment of CRC as well as screening modalities and the interpretation of screening results. The 2-wk follow-up module sent to trial enrollees in this arm included a welcome screen and a link to the National Cancer Institute’s CRC screening website. Recipients of this information were provided with links to the entire packets of information, regardless of relevance or preference.

### Data Sources and Measures

The primary outcome for the trial was receipt of CRC screening as documented in the EHR. We considered any documented receipt of CRC screening, regardless of physician recommendation or modality (i.e., colonoscopy or stool test), in the 12-mo period following the date of the office visit encounter that resulted in the patient’s trial eligibility.

In addition to compiling CRC screening receipt from the EHR, we also used data available within the EHR to obtain preenrollment characteristics of trial participants. These included patient age, race, sex, marital status, insurance coverage, Charlson Comorbidity Score,^50^ and whether English was the person’s preferred language.

Among those completing the pretrial survey, participants’ responses were used to further describe study participants at the time of enrollment and to evaluate hypothesized effect modification. Survey items included a measure of health literacy,^51^ CRC screening decision stage,^52^ decision-making preference,^53^ CRC-related worry,^54^ perceived CRC susceptibility,^55^ and CRC screening history.^56^

In addition to some broad measures of program reach (e.g., estimates of screening-eligible patients who do v. do not have active portal accounts) for which we have previously published results,^57^ we used the benchmark reporting function within the EHR to track two, more narrow, measures of reach among trial eligible patients. Among patients in the EHR registry (and thus due for CRC screening and with an activated portal account), we tracked the proportion who became study eligible by virtue of having a primary care visit with a CRC screening order and, among those, the proportions who opened the study messages sent to their portal inbox, interacted with the questionnaire attached to that message, and consented to study participation. These custom reports could be generated at any time by research staff, enabling real-time participant tracking. From these reports, we developed 2 measures to assess the degree to which the intervention or enhanced usual care material reached the study’s target population.^58^ First, among EHR registry patients (i.e., those of average risk and due for CRC screening with an activated portal account), we derived the proportion sent a secure message containing the intervention or enhanced usual care material, and second, among those sent a secure message, we derived the proportion who opened the message and thus saw that they were invited to the trial.

In addition to constructing these measures, for the individuals who consented to trial enrollment, we were able to compile additional information regarding implementation timing by using the date/time stamp associated with their interactions with study material. For example, we tracked the number of days between the office visit that triggered the person’s study eligibility (i.e., the date of CRC recommendation) and when the secure message that contained the study invitation was sent. Gaps in days between these 2 dates are due to routine variability in workflow processes, because not all physicians electronically sign the notes from each of their office visit encounters on the day of the visit. Second, we tracked the time in days between when a secure message was sent (i.e., the date the physician electronically signed the note) and when it was accessed by the patient in the online portal. Gaps in days between these 2 dates are due to routine variability in the frequency with which a person chooses to view secure messages and/or access his or her portal account.

### Statistical Analysis and Power

Continuous and categorical pretrial participant characteristics are summarized as mean (*s*) and counts (%), respectively. We estimated the effects of the characteristics on study arm assignment and intervention exposure. The effects of continuous, binary, and categorical characteristics were tested by Wilcoxon rank-sum test, Pearson chi-squared test, and Fisher exact test, respectively.

For the primary evaluation of effectiveness, we used an intent-to-treat (ITT) analysis among all people consenting to study participation. For the primary outcome, EHR-documented CRC screening receipt within 12 mo, we estimated the ITT effect by a 2-level hierarchical logistic regression model in which patients are nested within ordering physicians.^59^ As a secondary assessment of effectiveness, we limited the study sample to those trial enrollees who completed the pretrial survey and thus were exposed to the intervention or enhanced usual care material. With *N* = 1,800 patients seen by 150 physicians, we estimated having 0.86 power to detect a 7% change in CRC screening rates between the intervention and enhanced usual care arms (66% v. 59%, respectively).^43^ We also tested whether health literacy, decision stage, and decision-making preference moderated the intervention’s effectiveness, as planned by study protocol.^43^ The significance of effect moderation was tested by likelihood ratio tests. The latter could be tested only among those completing the baseline survey (i.e., the exposed sample).

## Results

### Study Participants

**Figure 1:**
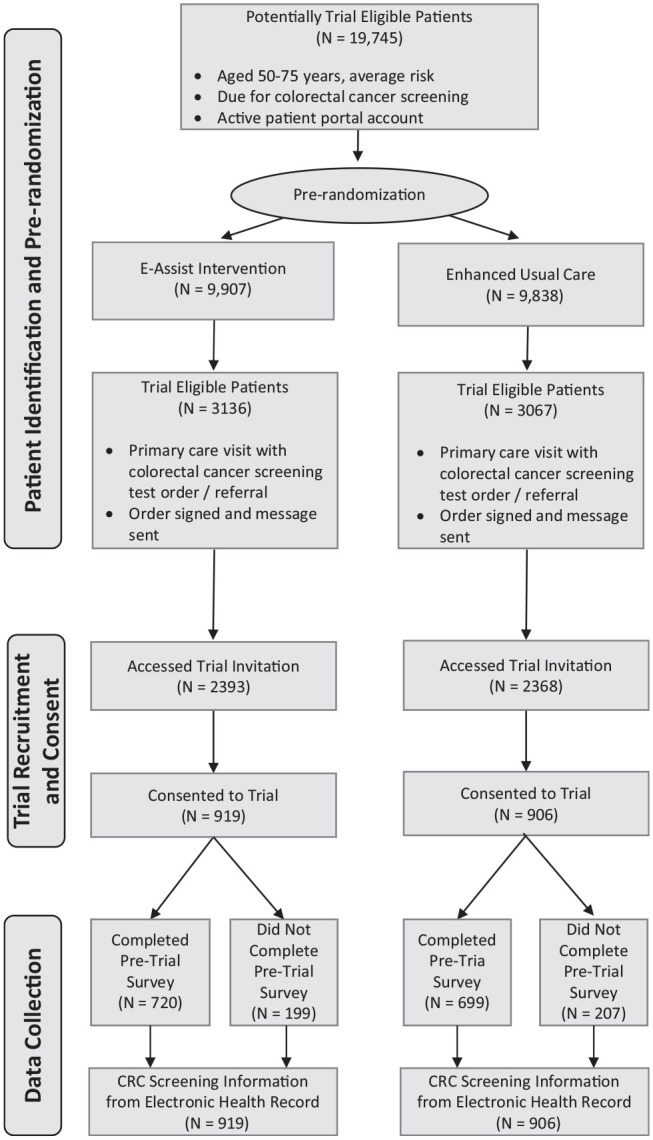
CONSORT flow diagram.

Between June 14, 2017, and September 15, 2019, 6203 people who were prerandomized to the e-Assist intervention or enhanced usual care trial arm had a primary care office visit with a physician order for CRC screening and thus became trial eligible (Figure 1). Among these, 4,761 accessed the trial invitation that was sent via patient portal message, and 1,825 consented to trial participation (919 in the e-Assist intervention arm and 906 in the enhanced usual care arm). A total of 170 primary care physicians electronically signed 1 or more of the CRC screening orders with a person who consented to trial participation.

On average, participants who consented to trial participation (*N* = 1,825; i.e., the ITT sample) were 59.7 y of age, 62.5% were female, and 30.1% were Black (Table 1). The proportion of trial enrollees who were married differed significantly by study arm, with 68.5% of those enrolled in the intervention arm married compared with 63.0% in enhanced usual care. In addition, those assigned to the intervention arm were significantly more likely to have a recommendation for colonoscopy screening (as opposed to stool testing; 83.4% v. 77.8%) at the time of trial enrollment. No other pretreatment differences were observed across the 2 study arms.

**Table 1:** Characteristics of Trial Participants by Study Arm (Intent-to-Treat Sample, *N* = 1825)

	e-Assist Intervention (*n* = 919)	Enhanced Usual Care (*n* = 906)	*P* Value
Continuous, $\bar{x}$ (*s*)			
Age, y	59.8 (7.3)	59.6 (7.2)	0.43
Charlson Comorbidity Score	1.1 (1.8)	1.1 (1.8)	0.58
Discrete, n (%)			
Race			0.69
White	545 (61.9)	540 (63.7)	
Black	273 (31.1)	247 (29.2)	
Other	63 (7.2)	60 (7.1)	
Gender			0.17
Female	560 (60.94)	580 (64.0)	
Male	359 (39.1)	326 (36.0)	
Insurance			0.81
Commercial	599 (67.2)	598 (66.1)	
Medicaid	25 (2.7)	21 (2.3)	
Medicare	287 (31.2)	275 (30.4)	
Other	8 (0.9)	11 (1.2)	
Marital status			0.01
Married	626 (68.5)	569 (63.0)	
Single	288 (31.5)	334 (37.0)	
Preferred language			0.63
English	889 (98.8)	851 (98.5)	
Other language	11 (1.2)	13 (1.5)	
Colorectal cancer screening order			<0.01
Colonoscopy	766 (83.4)	705 (77.8)	
Stool test only	153 (16.6)	201 (22.2)	

Among those consenting to trial participation, 1419 (78%) completed the baseline survey and were therefore exposed to the intervention/enhanced usual care material. This included 720 (78.3%) of the patients in the intervention arm and 699 (77.2%) of the patients in the enhanced usual care arm. With the exception that women who consented to trial participation were more likely to go on to complete the baseline survey and therefore be exposed to the intervention/enhanced usual care material, we did not find statistically significant differences among those who consented to trial participation by exposure status (Supplementary Appendix Table A-1.).

As within the ITT sample, among the exposed sample, pretrial characteristics did not differ significantly across study arms, except for marital status (68.6% married in the e-Assist arm v. 60.9% married in the enhanced usual care arm) and receipt of a colonoscopy referral, which remained significantly higher in those allocated to the intervention compared with enhanced usual care (84.2% v. 77.3%, respectively; data not shown). Pretrial survey responses among the exposed sample did not differ by study arm (Table 2).

**Table 2:** Pretrial Survey Responses among the Exposed Sample (*N* = 1419) by Study Arm

Variable	e-Assist Intervention (*n* = 720)	Enhanced Usual Care (*n* = 699)	*P* Value
Health literacy			0.59
Not confident	70 (9.8%)	60 (8.6%)	
Somewhat confident	184 (25.6%)	192 (27.7%)	
Quite confident	464 (64.6%)	442 (63.7%)	
Ever had CRC screening			0.59
Yes	505 (70.3%)	478 (68.9%)	
No	213 (29.7%)	216 (31.1%)	
Worried about CRC			0.16
Not at all/slightly	503 (70.1%)	453 (65.3%)	
Somewhat	128 (17.8%)	142 (20.4%)	
Moderately/extremely	87 (12.1%)	99 (14.3%)	
Decision-making preference			0.78
Prefer to make decision	433 (60.3%)	429 (61.8%)	
Prefer share with doctor	213 (29.7%)	194 (28.0%)	
Prefer doctor to make	72 (10.1%)	71 (10.2%)	
Perceived CRC susceptibility			0.88
Low	567 (79.0%)	553 (79.7%)	
Moderate	137 (19.1%)	126 (18.2%)	
High	14 (1.9%)	15 (2.1%)	
Screening decision stage			0.53
Screened in past 6 mo	18 (2.5%)	21 (3.0%)	
Decided to screen, <6 mo	591 (82.3%)	558 (80.4%)	
Decided to screen, >6 mo	29 (4.0%)	30 (4.3%)	
Undecided regarding screening	70 (9.7%)	67 (9.7%)	
Decided against screening	10 (1.5%)	18 (2.6%)	

CRC, colorectal cancer.

### Intervention Effectiveness

Sixty-five percent (65.1%) of trial enrollees who consented were screened for CRC in the 12 mo following the office visit that triggered their trial eligibility (i.e., a primary care office visit with a CRC screening recommendation), 65.7% in the intervention and 64.5% in the enhanced usual care arm. Most of these screenings occurred within the first 6 mo after that office visit: 47.1% of trial enrollees were screened within 3 mo of their office visit and 60.1% within 6 mo of their office visit. This is in comparison with an overall CRC screening rate of 62% among the health system’s general population. Among those exposed to either the intervention or enhanced usual care material, screening rates were 67.8% in the intervention-exposed group and 66.7% in the enhanced usual care exposed group. Most screenings within the exposed groups similarly occurred within 6 mo of their office visit, 49.8% within 3 mo of their visit, and 62.2% within 6 mo. None of these differences were statistically significant (i.e., *P* > .10), nor were 12-mo screening rates (the study’s primary outcome) statistically different once we adjusted for pretreatment covariates that differed significantly across the 2 study arms (Table 3).

**Table 3:** Adjusted Logistic Regression Results for Intervention Effect on Colorectal Cancer Screening: Intent-to-Treat (*n* = 1825) and Exposed (*n* = 1419) Samples

	Intent-to-Treat Sample		Exposed Sample	
	Odds Ratio	95% Confidence Interval	Odds Ratio	95% Confidence Interval
Intercept	—		—	
Intervention	1.1	0.6–1.8	0.9	0.5–1.7
Currently married	1.7	1.3–2.3	1.8	1.3–2.5
Intervention × currently married	0.8	0.6–1.3	0.8	0.5–1.3
Referral for colonoscopy testing	0.8	0.6–1.2	0.7	0.5–1.0
Intervention × colonoscopy referral	1.1	0.7–1.9	0.3	0.8–2.5
τ	0.1		0.1	
ICC^a^	0.04		0.03	

a Variance of physician-specific random effect; ICC, intraphysician correlation coefficient $\tau /(\tau +\pi^{2}/3)$.

In addition, within the exposed sample, we found no statistically significant effect moderation by any of the 3 a priori hypothesized patient characteristics (Table 4). It should be noted, however, that 83.9% of participants reported either having been screened (2.9%) or intending to be screened for CRC in the next 6 mo (81.0%) at the time they completed the baseline survey (i.e., before being exposed to the intervention or enhanced usual care material; Table 2). Similarly, almost two-thirds of exposed participants endorsed being of high health literacy and preferring to be responsible for making decisions regarding screening (Table 2).

**Table 4:** Adjusted Logistic Regression Results for Intervention Moderating Effects on Colorectal Cancer Screening: Exposed Sample (*n* = 1419)

	Odds Ratio	95% Confidence Interval
Model with health literacy		
Intercept	—	
Intervention	0.7	0.3–1.7
Health literacy		
Not confident	1.0	
Somewhat confident	0.5	0.2–1.0
Quite confident health	0.4	0.2–0.8
Intervention × literacy		
Not confident	1.0	
Somewhat confident	1.5	0.6–4.1
Quite confident	1.6	0.6–3.9
τ	0.1	
ICC^a^	0.03	
Model with decision-making preference		
Intercept	—	
Intervention	1.2	0.9–1.6
Decision-making preference		
Prefer to make	1.0	
Share with doctor	1.4	0.9–2.0
Prefer doctor make	1.6	0.9–2.8
Intervention × decision preference		
Prefer to make	1.0	
Share with doctor	0.8	0.5–1.4
Prefer doctor make	0.6	0.3–1.2
τ	0.1	
ICC	0.03	
Model with Decision stage		
Intercept	—	
Intervention	0.4	0.1–2.3
Decision stage^b^		
Screened in past 6 mo	1.0	
Decided to screen, <6 mo	0.2	0.1–1.0
Decided to screen, >6 mo	0.1	0.0–0.5
Intervention × decision stage		
Screened in past 6 mo	1.0	
Decided to screen, <6 mo	3.0	0.5–19.6
Decided to screen, >6 mo	2.8	0.4–19.1
τ	0.1	
ICC	0.03	

a Variance of physician-specific random effect; ICC, intraphysician correlation coefficient $\tau /(\tau +\pi^{2}/3)$. To enable model convergence, the decision stage was grouped into 3 categories (1: screened within the past 6 mo, 2: intent to screen within 6 mo, and 3: otherwise [i.e., intent to screen, but not within 6 mo; undecided regarding screening; or decided against screening]).

### Intervention Implementation

**Figure 2:**
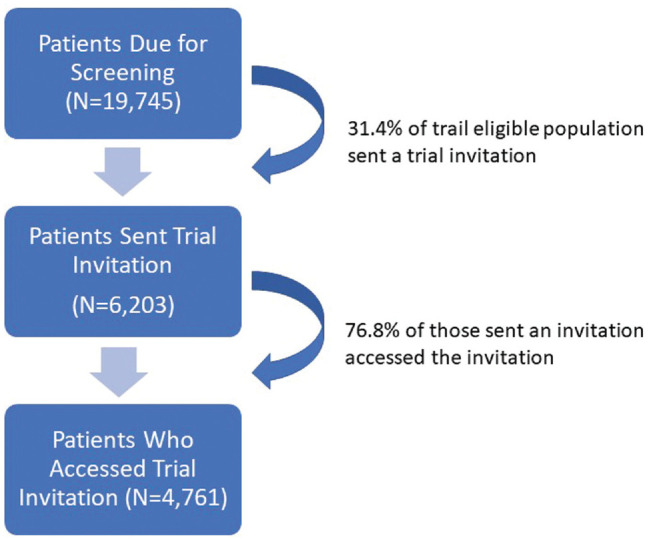
Program reach among trial-eligible patients.

We randomized a total of *N* = 19,745 people who, per data contained in the EHR, were insured, aged 50 to 75 y with an activated online portal account, and of average risk and due for CRC screening. Between June 14, 2017, and September 15, 2019, almost one-third (*n* = 6,203 or 31.4%) became study eligible because they had a primary care visit with a recommendation for CRC screening. Among those sent a message, more than three-quarters (*n* = 4761 or 76.8%) accessed the trial invitation. Overall, this resulted in the intervention reaching approximately one-quarter (24.1%) of the study-eligible patients (Figure 2).

**Figure 3:**
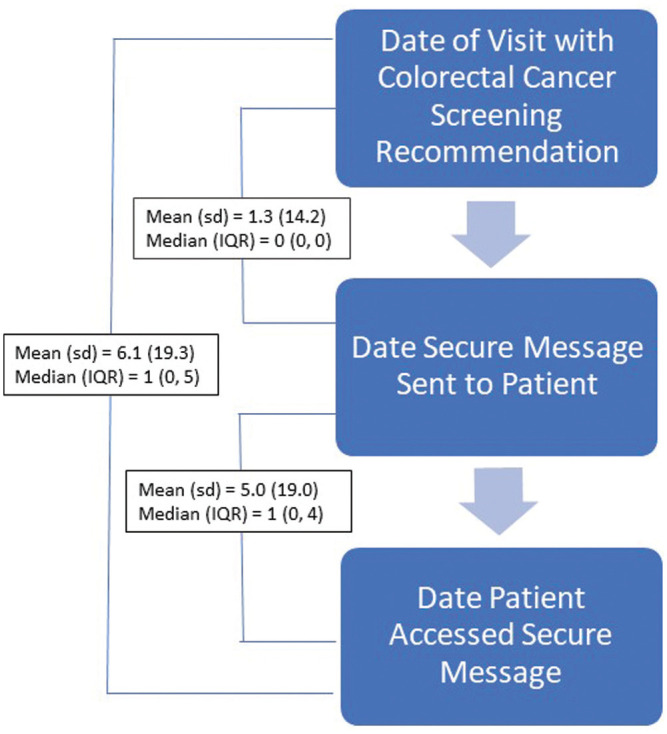
Timeliness of intervention activities among trial-enrolled participants.

Among study-eligible people who accessed the trial invitation, 38.3% enrolled in the trial (*N* = 1825), enabling us to meet study enrollment goals. Those who enrolled in the trial accessed the secure message inviting them to participate in the trial at an average of 6.1 (*s* = 19.3) d after the primary care visit in which they received a recommendation for CRC screening (Figure 3). This time delay is a result of both physician and patient actions. First, the secure message inviting the patient to the trial was sent, on average, 1.3 (*s* = 14.1) d following the patient’s office visit (i.e., the average time between an office visit and the physician electronically signing the office visit note). While this ranged from the same day to 586 d after the visit, 74% of visit notes were signed within 4 d, and only 61 patients (3.3%) were sent the secure message more than 7 d after their appointment. Second, once a secure message was sent, it was accessed by the patient on average 5.0 d later, although this too ranged widely (from 0 to 352 d). This resulted in a handful of patients (*n* = 69) consenting to trial participation after they completed CRC screening. In addition, some trial enrollees (*n* = 44) consented to trial participation without the study team capturing a message read date. This happened either because the patient used the questionnaire page within their portal to access the questionnaire that contained the trial consent, baseline survey, and intervention/enhanced usual care material (as opposed to using the attachment provided with the study invitation secure message) or because the message and/or questionnaire were accessed via a linked proxy account. None of these nuanced situations differed significantly by study arm. Nor did accounting for them alter the effectiveness results or conclusions (data not shown).

## Discussion

By leveraging the functionality of an EHR, we were able to automate both the identification of patients and the delivery of an intervention designed to engage and support patients to complete CRC screening after a physician recommended it. Doing so ensured the intervention’s integration with existing primary care workflows without requiring busy primary care providers and staff to alter current practices. Despite this successful practice integration, patients’ use of the intervention did not improve CRC screening rates. This may have been at least in part because the use of an EHR-tethered patient portal limited our ability to reach those patients for whom the intervention may have been most effective. This latter group, for example, may include those who are aware that screening is recommended, have an interest in learning more, but have not yet made a commitment to acting, or those who may want to act, but who may face challenges in following through on their doctor’s recommendation for care. As such, our results highlight some of the current challenges in improving CRC screening rates among primary care patients as well as both the opportunities and challenges in using the functionalities of the EHR to do so.

Importantly, given providers’ interest in leveraging their investments in EHR technology, we found that using the EHR enabled us to efficiently identify and automate the sending of a patient-facing intervention following a physician recommendation for care. While this ability is potentially important to integrating patient-facing interventions into clinical care delivery, there are significant limitations in such interventions reaching patients when relying on patient portals as the sole mode of delivery. Using data from this study’s sampling process, we have previously shown how only 28% of adults aged 50 to 75 y who are due for CRC screening have an activated portal account as well as how, among this population, Black adults are substantively and significantly less likely to have an active portal account.^57^ Thus, although we ultimately reached only a quarter of study-eligible patients, we reached a substantially smaller proportion of all primary care patients due for CRC screening, and those we did not reach were disproportionately Black.

Furthermore, as indicated by responses to the pretrial survey, those reached by the intervention reported generally high levels of health literacy, a strong preference to make their own decisions regarding CRC screening, and a strong intent to be screened for CRC at the time of study enrollment. This selectivity is consistent with prior studies that found an association between primary care visit frequency^60^ and CRC screening as well as an association between cancer screening and portal use.^61^ Multiple studies that have been published since this intervention was conceived have shown how Black and racially or otherwise minoritized adults are not only less likely to use the patient portal^62–66^ but also to use each aspect of the portal less.^67,68^ As such, relying on patient portals alone to reach patients due for CRC screening (or any other service) may not only limit the effectiveness of the intervention but also further contribute to existing disparities and exacerbate existing structural racism.

Despite the limitations in overall reach when using portals for patient-facing decision support, our results illustrate how EHR-tethered portals can be used as a low-cost means to engage certain patients. In this study, more than three-quarters of patients who were sent a secure message via their online portal not only accessed that message but did so in a timely manner (i.e., within 1 wk of their office visit). This finding is consistent with those of others researchers who successfully engaged portal users around the time of a scheduled visit to primary care.^69^ Thus, for the subset of patients with an active portal account, secure messaging around the time of an office visit may be a good way to route supplemental material to patients that addresses common deficits in physician office-based recommendations for care. But given known racial disparities in portal use, including within the context of this trial,^57^ such interventions must be used in combination with other modalities such as telephone calls, text messages, and/or print material. Regardless of modality, ongoing challenges integrating decision aids and shared decision making more generally into practice highlight the importance of continuing to explore how not only to embed patient-facing interventions within clinic workflows but how to do so in ways that reach all of those needing support. Doing so undoubtably will mean not relying on one communication channel but instead devising ways to meet patients where they are most comfortable and able to receive that support.

Regardless of challenges in intervention reach, for those reached, the provision of a theory-based intervention designed to complement physician office-based recommendations for CRC screening did not result in more screening than simply providing links to existing online educational material on CRC etiology, symptoms, screening, and treatment. Importantly, more than 80% of the people reached indicated that they had either been screened in the prior 6 mo or intended to be screened in the next 6 mo before they ever interacted with the intervention, and screening use among these people topped 75%. While the intervention may be relatively more effective in supporting screening among those who had less intentions to be screened, the portal-based intervention did not frequently reach such people. Whether the same intervention content might be effective among those still contemplating whether to be screened or whether a more targeted intervention that focused more heavily on barrier removal might be effective among the population reached here should be explored in the future.

Because few studies have reported how complex EHR functionalities such as BPAs, registries, and health maintenance flags can be used to support research activities,^70–72^ our findings contribute to the literature on the use of EHRs in pragmatic trials and other types of practice-embedded research. While, as evidenced by our findings, caution needs to exist surrounding the sole reliance on portals to route patient-facing material to people,^57^ it is important to note that the other EHR functionalities we leveraged work independently of the portal. In addition, most of the functionalities can be set to work silently, in the background of the EHR, and thus in ways that remain not visible or disruptive to clinic staff. Patient identification, follow-up, and even the collection of patient-reported outcomes via in-clinic use of such EHR-embedded tools^73^ can therefore greatly facilitate pragmatic and other trial processes.

Study limitations include the sole reliance on EHR data to obtain CRC screening information, as such a reliance may have led to underreporting of CRC use. We do not, however, have reason to believe that this underreporting would have differed by study arm. As noted above, given the reliance on the patient portal, a primary study limitation was the limited ability to reach the targeted population. In addition to the challenges reaching the target population, among those who viewed the secure study invitation, there may have been additional selectivity bias, as enrolling in the trial required the ability not only to get to the secure message but open the attachment and navigate within the questionnaire. This may have been especially true for patients who prefer to receive information in a language other than English. In addition, it is possible that those consenting to study participation differed from those who may otherwise elect to engage with similar interventions outside of a research setting. It may also be that physicians selectively order CRC screening for only those patients who express an interest during the office visit (or, at least, selectively do not order screening for those verbally refusing screening), further limiting the reach of our intervention. Regardless, an intervention tied to an office-based physician order for care will never reach those who choose not to seek care, highlighting the importance of both in-reach and outreach strategies to improve CRC screening rates in the primary care setting. Because our study did not collect reasons for nonenrollment among those who did not consent to study participation, we are not able to shed light on why people may have elected not to view the study material. Furthermore, without a sample of people who provided consent but were not exposed to anything (i.e., true usual care), we are not able to determine whether the simple provision of information on CRC and CRC screening might improve CRC screening use following a physician recommendation for care. Given what is known about the complexities of behavior change and that the CRC screening rates observed among trial enrollees (65%) were approximately equal to those observed among the health system’s general population of primary care patients aged 50 to 75 y with an activated portal account around the same time (62%), this would seem highly unlikely. There may also be practice-level factors such as scheduling delays or the use of reminder calls, which could affect CRC screening timeliness and ultimately use. Although we did not compile such information within the context of this trial, most practice-level preventive health initiatives are conducted by a centralized (i.e., health system-level) population health management team within the health system within which the study was conducted, thus minimizing potential confounding.

## Conclusions

While embedding patient-facing interventions within the EHR enables much needed practice integration, doing so likely minimizes program effectiveness by presenting challenges in reaching important segments of the patient population and may even exacerbate well-known racial and other disparities.

## Supplemental Material

sj-docx-1-mdm-10.1177_0272989X221104094 – Supplemental material for Opportunities and Challenges When Using the Electronic Health Record for Practice-Integrated Patient-Facing Interventions: The e-Assist Colon Health Randomized Trial Click here for additional data file. Supplemental material, sj-docx-1-mdm-10.1177_0272989X221104094 for Opportunities and Challenges When Using the Electronic Health Record for Practice-Integrated Patient-Facing Interventions: The e-Assist Colon Health Randomized Trial by Jennifer Elston Lafata, Deirdre A. Shires, Yongyun Shin, Susan Flocke, Kenneth Resnicow, Morgan Johnson, Ellen Nixon, Xinxin Sun and Sarah Hawley in Medical Decision Making

## References

[1] American Cancer Society. Colorectal Cancer Facts and Figures 2020-2022. Atlanta (GA): American Cancer Society; 2020.

[2] Lafata JE Cooper G Divine G Oja-Tebbe N Flocke SA . Patient-physician colorectal cancer screening discussion content and patients’ use of colorectal cancer screening. Patient Educ Couns. 2014;94(1):76–82. DOI: 10.1016/j.pec.2013.09.008 24094919 PMC3865022

[3] Lafata JE Divine G Moon C Williams LK . Patient-physician colorectal cancer screening discussions and screening use. Am J Prev Med. 2006;31(3):202–9. DOI: 10.1016/j.amepre.2006.04.010 PMC4682196 16905030

[4] Hoffman RM Lewis CL Pignone MP , et al. Decision-making processes for breast, colorectal, and prostate cancer screening: the DECISIONS survey. Med Decis Making. 2010;30(5 suppl):53S–64S. DOI: 0272989X10378701 20881154 10.1177/0272989X10378701 PMC3139436

[5] Cairns CP Viswanath K . Communication and colorectal cancer screening among the uninsured: data from the Health Information National Trends Survey (United States). Cancer Causes & Control. 2006;17(9):1115–25. 10.1007/s10552-006-0046-2 17006717

[6] Feeley TH Cooper J Foels T Mahoney MC . Efficacy expectations for colorectal cancer screening in primary care: identifying barriers and facilitators for patients and clinicians. Health Commun. 2009;24(4):304–15. DOI: 10.1080/10410230902889241 19499424

[7] Fenton JJ Jerant AF von Friederichs-Fitzwater MM Tancredi DJ Franks P . Physician counseling for colorectal cancer screening: Impact on patient attitudes, beliefs, and behavior. J Am Board Fam Med. 2011;24(6):673–81. DOI: 10.3122/jabfm.2011.06.110001 22086810

[8] Katz ML James AS Pignone MP , et al. Colorectal cancer screening among African American church members: a qualitative and quantitative study of patient-provider communication. BMC Public Health. 2004;4:62. DOI: 1471-2458-4-62 15601463 10.1186/1471-2458-4-62 PMC544572

[9] Lafata JE Cooper GS Divine G , et al. Patient-physician colorectal cancer screening discussions: delivery of the 5A’s in practice. Am J Prev Med. 2011;41(5):480–6. DOI: 10.1016/j.amepre.2011.07.018 PMC4657138 22011418

[10] Ling BS Klein WM Dang Q . Relationship of communication and information measures to colorectal cancer screening utilization: results from HINTS. J Health Commun. 2006;(1 suppl 1):181–90. DOI: 10.1080/10810730600639190 16641083

[11] Ling BS Trauth JM Fine MJ , et al. Informed decision-making and colorectal cancer screening: is it occurring in primary care? Med Care. 2008;46(9 suppl 1):S23–9. DOI: 10.1097/MLR.0b013e31817dc496 18725829

[12] McQueen A Bartholomew LK Greisinger AJ , et al. Behind closed doors: physician-patient discussions about colorectal cancer screening. J Gen Intern Med. 2009;24(11):1228–35. DOI: 10.1007/s11606-009-1108-4 PMC2771240 19763699

[13] Wackerbarth SB Tarasenko YN Joyce JM Haist SA . Physician colorectal cancer screening recommendations: an examination based on informed decision making. Patient Educ Couns. 2007;66(1):43–50. DOI: 10.1016/j.pec.2006.10.003 17098393 PMC3635666

[14] Walsh JM Karliner L Burke N Somkin CP Pham LA Pasick R . Physicians’ approaches to recommending colorectal cancer screening: a qualitative study. J Cancer Educ. 2010;25(3):385–90. DOI: 10.1007/s13187-010-0058-1 PMC2936570 20204571

[15] Wunderlich T Cooper G Divine G , et al. Inconsistencies in patient perceptions and observer ratings of shared decision making: the case of colorectal cancer screening. Patient Educ Couns. 2010;80(3):358–63. DOI: S0738-3991(10)00396-4 10.1016/j.pec.2010.06.034 PMC2971658 20667678

[16] O’Farrell CM Green BB Reid RJ Bowen D Baldwin LM . Physician-patient colorectal cancer screening discussions by physicians’ screening rates. J Am Board Fam Med. 2012;25(6):771–81. 10.3122/jabfm.2012.06.110279 23136315

[17] Flocke SA Stange KC Cooper GS , et al. Patient-rated importance and receipt of information for colorectal cancer screening. Cancer Epidemiol Biomarkers Prev. 2011;20(10):2168–73. DOI: 1055-9965.EPI-11-0281 10.1158/1055-9965.EPI-11-0281 PMC3189279 21813727

[18] Holden DJ Jonas DE Porterfield DS Reuland D Harris R . Systematic review: enhancing the use and quality of colorectal cancer screening. Ann Intern Med. 2010;152(10):668–76. DOI: 10.7326/0003-4819-152-10-201005180-00239 20388703

[19] Lairson DR Dicarlo M Deshmuk AA , et al. Cost-effectiveness of a standard intervention versus a navigated intervention on colorectal cancer screening use in primary care. Cancer. 2014;120(7):1042–9. DOI: 10.1002/cncr.28535 PMC3961516 24435411

[20] O’Connor AM Bennett CL Stacey D , et al. Decision aids for people facing health treatment or screening decisions. Cochrane Database Syst Rev. 2009;(3):CD001431. 10.1002/14651858.CD001431.pub2 19588325

[21] Stacey D Bennett CL Barry MJ , et al. Decision aids for people facing health treatment or screening decisions. Cochrane Database Syst Rev. 2011;(10):CD001431. 10.1002/14651858.CD001431.pub3 21975733

[22] Stacey D Légaré F Col NF , et al. Decision aids for people facing health treatment or screening decisions. Cochrane Database Syst Rev. 2014;(1):CD001431. 10.1002/14651858.CD001431.pub4 24470076

[23] Jimbo M Rana GK Hawley S , et al. What is lacking in current decision aids on cancer screening? CA Cancer J Clin. 2013;63(3):193–214. 23504675 10.3322/caac.21180 PMC3644368

[24] Volk RJ Linder SK Lopez-Olivo MA , et al. Patient decision aids for colorectal cancer screening: a systematic review and meta-analysis. Am J Prev Med. 2016;51(5):779–91. DOI: 10.1016/j.amepre.2016.06.022 PMC5067222 27593418

[25] Graham ID Logan J O’Connor A , et al. A qualitative study of physicians’ perceptions of three decision aids. Patient Educ Couns. 2003;50(3):279–83. 10.1016/s0738-3991(03)00050-8 12900100

[26] Brace C Schmocker S Huang H Victor JC McLeod RS Kennedy ED . Physicians’ awareness and attitudes toward decision aids for patients with cancer. J Clin Oncol. 2010;28(13):2286–92. DOI: 10.1200/JCO.2009.25.2874 20354133

[27] Zeliadt SB Hoffman RM Birkby G , et al. Challenges implementing lung cancer screening in federally qualified health centers. Am J Prev Med. 2018;54(4):568–75. DOI: 10.1016/j.amepre.2018.01.001 PMC8483158 29429606

[28] Elwyn G Scholl I Tietbohl C , et al. “Many miles to go…”: a systematic review of the implementation of patient decision support interventions into routine clinical practice. BMC Med Inform Decis Making. 2013;13(suppl 2):S14. DOI: 10.1186/1472-6947-13-S2-S14 PMC4044318 24625083

[29] Sabatino SA Lawrence B Elder R , et al. Effectiveness of interventions to increase screening for breast, cervical, and colorectal cancers nine updated systematic reviews for the guide to community preventive services. Am J Prev Med. 2012;43(1):97–118. DOI: 10.1016/j.amepre.2012.04.009 22704754

[30] Basch CE Wolf RL Brouse CH , et al. Telephone outreach to increase colorectal cancer screening in an urban minority population. Am J Public Health. 2006;96(12):2246–53. DOI: AJPH.2005.067223 10.2105/AJPH.2005.067223 PMC1698159 17077394

[31] Dietrich AJ Tobin JN Cassells A , et al. Telephone care management to improve cancer screening among low-income women: a randomized, controlled trial. Ann Intern Med. 2006;144(8):563–71. DOI: 144/8/563 10.7326/0003-4819-144-8-200604180-00006 PMC3841972 16618953

[32] Myers RE Ross E Jepson C , et al. Modeling adherence to colorectal cancer screening. Prev Med. 1994;23(2):142–51. DOI: S0091-7435(84)71020-6 10.1006/pmed.1994.1020 8047519

[33] Myers RE Ross EA Wolf TA Balshem A Jepson C Millner L . Behavioral interventions to increase adherence in colorectal cancer screening. Med Care. 1991;29(10):1039–50. 10.1097/00005650-199110000-00009 1921523

[34] Goldberg D Schiff GD McNutt R Furumoto-Dawson A Hammerman M Hoffman A . Mailings timed to patients’ appointments: a controlled trial of fecal occult blood test cards. Am J Prev Med. 2004;26(5):431–5. DOI: 10.1016/j.amepre.2004.02.009S0749379704000364 15165660

[35] Church TR Yeazel MW Jones RM , et al. A randomized trial of direct mailing of fecal occult blood tests to increase colorectal cancer screening. J Natl Cancer Inst. 2004;96(10):770–80. 10.1093/jnci/djh134 15150305

[36] Hardcastle JD Armitage NC Chamberlain J Amar SS James PD Balfour TW . Fecal occult blood screening for colorectal cancer in the general population: results of a controlled trial. Cancer. 1986;58(2):397–403. 3719535 10.1002/1097-0142(19860715)58:2<397::aid-cncr2820580235>3.0.co;2-x

[37] Mosen DM Feldstein AC Perrin N , et al. Automated telephone calls improved completion of fecal occult blood testing. Med Care. 2010;48(7):604–10. DOI: 10.1097/MLR.0b013e3181dbdce7 PMC3738295 20508529

[38] Hardcastle JD Chamberlain JO Robinson MH , et al. Randomised controlled trial of faecal-occult-blood screening for colorectal cancer. Lancet. 1996;348(9040):1472–7. DOI: S0140-6736(96)03386-7 10.1016/S0140-6736(96)03386-7 8942775

[39] Baron RC Mercer SL Se TFCP . Recommendations for client- and provider-directed interventions to increase breast, cervical, and colorectal cancer screening. Am J Prev Med. 2008;35(1):S21–5. DOI: 10.1016/j.amepre.2008.04.004 18541184

[40] Baron RC Rimer BK Breslow RA , et al. Client-directed interventions to increase community demand for breast, cervical, and colorectal cancer screening. Am J Prev Med. 2008;35(1):S34–55. DOI: 10.1016/j.amepre.2008.04.002 18541187

[41] Tabriz AA Flocke SA Shires D Dyer KE Schreiber M Elston Lafata J . Logic model framework for considering the inputs, processes and outcomes of a healthcare organisation-research partnership. BMJ Qual Saf. 2020;29(9):746–55. DOI: 10.1136/bmjqs-2019-010059 PMC8675565 31826921

[42] Lin JS Piper MA Perdue LA , et al. Screening for Colorectal Cancer: A Systematic Review for the US Preventive Services Task Force. Rockville (MD): US Preventive Services Task Force; 2016. 27441328

[43] Lafata JE Shin Y Flocke SA , et al. Randomised trial to evaluate the effectiveness and impact of offering postvisit decision support and assistance in obtaining physician-recommended colorectal cancer screening: the e-assist: colon health study—a protocol study. BMJ Open. 2019;9(1):e023986. DOI: 10.1136/bmjopen-2018-023986 PMC6326296 30617102

[44] Janz NK Becker MH . The health belief model: a decade later. Health Educ Q. 1984;11(1):1–47. DOI: 10.1177/109019818401100101 6392204

[45] Deci EL Koestner R Ryan RM . A meta-analytic review of experiments examining the effects of extrinsic rewards on intrinsic motivation. Psychol Bull. 1999;125(6):627–68. DOI: 10.1037/0033-2909.125.6.627 10589297

[46] Weinstein ND Lyon JE Sandman PM Cuite CL . Experimental evidence for stages of health behavior change: the precaution adoption process model applied to home radon testing. Health Psychol. 1998;17(5):445–53. DOI: 10.1037//0278-6133.17.5.445 9776003

[47] Resnicow K McMaster F . Motivational Interviewing: moving from why to how with autonomy support. Int J Behav Nutr Phys Act. 2012;9:19. DOI: 10.1186/1479-5868-9-19 22385702 PMC3330017

[48] Resnicow K Zhou Y Hawley S , et al. Communication preference moderates the effect of a tailored intervention to increase colorectal cancer screening among African Americans. Patient Educ Couns. 2014;97(3):370–5. DOI: 10.1016/j.pec.2014.08.013 PMC6208142 25224317

[49] Healthwise. Healthwise—for every health decision. Colorectal cancer. 2018. Available from: http://www.healthwise.net

[50] Deyo RA Cherkin DC Ciol MA . Adapting a clinical comorbidity index for use with ICD-9-CM administrative databases. J Clin Epidemiol. 1992;45(6):613–9. DOI: 10.1016/0895-4356(92)90133-8 1607900

[51] Chew LD Griffin JM Partin MR , et al. Validation of screening questions for limited health literacy in a large VA outpatient population. J Gen Intern Med. 2008;23(5):561–6. DOI: 10.1007/s11606-008-0520-5 PMC2324160 18335281

[52] Sifri R Rosenthal M Hyslop T , et al. Factors associated with colorectal cancer screening decision stage. Prev Med. 2010;51(3–4):329–31. DOI: 10.1016/j.ypmed.2010.06.015 20600255

[53] Degner LF Sloan JA Venkatesh P . The Control Preferences Scale. Can J Nurs Res. 1997;29(3):21–43. 9505581

[54] National Cancer Institute. Health information national trends survey cycle 4. 2014. February 2018. Available from: https://hints.cancer.gov/docs/Instruments/HINTS-4_Cycle_4_English_Annotated_Form.pdf

[55] McQueen A Tiro JA Vernon SW . Construct validity and invariance of four factors associated with colorectal cancer screening across gender, race, and prior screening. Cancer Epidemiol Biomarkers Prev. 2008;17(9):2231–7. DOI: 10.1158/1055-9965.EPI-08-0176 PMC2603464 18768488

[56] National Cancer Institute. Health information national trends survey 3 2008. February 2018. Available from: https://hints.cancer.gov/docs/Instruments/HINTS2007FINALREPORT.pdf

[57] Tabriz AA Fleming PJ Shin Y , et al. Challenges and opportunities using online portals to recruit diverse patients to behavioral trials. J Am Med Inform Assoc. 2019;26(12):1637–44. DOI: 10.1093/jamia/ocz157 PMC6857600 31532482

[58] Glasgow RE Vogt TM Boles SM . Evaluating the public health impact of health promotion interventions: the RE-AIM framework. Am J Public Health. 1999;89(9):1322–7. DOI: 10.2105/ajph.89.9.1322 PMC1508772 10474547

[59] Raudenbush SW BA . Hierarchical Linear Models. Newbury Park (CA): Sage; 2002.

[60] Shires DA Divine G Schum M , et al. Colorectal cancer screening use among insured primary care patients. Am J Manag Care. 2011;17(7):480–8. 21819168

[61] Woolf SH Krist AH Lafata JE , et al. Engaging patients in decisions about cancer screening: exploring the decision journey through the use of a patient portal. Am J Prev Med. 2018;54(2):237–47. DOI: 10.1016/j.amepre.2017.10.027 PMC7144024 29241715

[62] Irizarry T DeVito Dabbs A Curran CR . Patient portals and patient engagement: a state of the science review. J Med Internet Res. 2015;17(6):e148. DOI: 10.2196/jmir.4255 PMC4526960 26104044

[63] Smith SG O’Conor R Aitken W Curtis LM Wolf MS Goel MS . Disparities in registration and use of an online patient portal among older adults: findings from the LitCog cohort. J Am Med Inform Assoc. 2015;22(4):888–95. DOI: 10.1093/jamia/ocv025 PMC4810779 25914099

[64] Griffin A Skinner A Thornhill J Weinberger M . Patient portals: who uses them? What features do they use? And do they reduce hospital readmissions? Appl Clin Inform. 2016;7(2):489–501. DOI: 10.4338/ACI-2016-01-RA-0003 27437056 PMC4941855

[65] Peacock S Reddy A Leveille SG , et al. Patient portals and personal health information online: perception, access, and use by US adults. J Am Med Inform Assoc. 2017;24(e1):e173–7. DOI: 10.1093/jamia/ocw095 PMC7651932 27413120

[66] Wedd J Basu M Curtis LM , et al. Racial, ethnic, and socioeconomic disparities in web-based patient portal usage among kidney and liver transplant recipients: cross-sectional study. J Med Internet Res. 2019;21(4):e11864. DOI: 10.2196/11864 PMC6658258 31008707

[67] Elston Lafata J Miller CA Shires DA Dyer K Ratliff SM Schreiber M . Patients’ adoption of and feature access within electronic patient portals. Am J Manag Care. 2018;24(11):e352–7. PMC6613379 30452203

[68] Price-Haywood EG Luo Q Monlezun D . Dose effect of patient-care team communication via secure portal messaging on glucose and blood pressure control. J Am Med Inform Assoc. 2018;25(6):702–8. DOI: 10.1093/jamia/ocx161 PMC7647025 29444256

[69] Dharod A Bellinger C Foley K Case LD Miller D . The reach and feasibility of an interactive lung cancer screening decision aid delivered by patient portal. Appl Clin Inform. 2019;10(1):19–27. DOI: 10.1055/s-0038-1676807 30625501 PMC8438623

[70] Devoe C Gabbidon H Schussler N , et al. Use of electronic health records to develop and implement a silent best practice alert notification system for patient recruitment in clinical research: quality improvement initiative. JMIR Med Inform. 2019;7(2):e10020. DOI: 10.2196/10020 PMC6658304 31025947

[71] Embi PJ Jain A Clark J Bizjack S Hornung R Harris CM . Effect of a clinical trial alert system on physician participation in trial recruitment. Arch Intern Med. 2005;165(19):2272–7. DOI: 10.1001/archinte.165.19.2272 PMC1343501 16246994

[72] Tan MH Bernstein SJ Gendler S Hanauer D Herman WH . Design, development and deployment of a Diabetes Research Registry to facilitate recruitment in clinical research. Contemp Clin Trials. 2016;47:202–8. DOI: 10.1016/j.cct.2016.01.010 PMC4818158 26825022

[73] Sinha S Garriga M Naik N , et al. Disparities in electronic health record patient portal enrollment among oncology patients. JAMA Oncol. 2021;7(6):935–7. DOI: 10.1001/jamaoncol.2021.0540 PMC8033503 33830178

