# Supplementary material for: Opportunities and Challenges When Using the Electronic Health Record for Practice-Integrated Patient-Facing Interventions: The e-Assist Colon Health Randomized Trial
Source: Med Decis Making. 2022 Jun 28;42(8):985–98. doi: 10.1177/0272989X221104094 (PMC9583291; doi:10.1177/0272989X221104094)
Supplement: sj-docx-1-mdm-10.1177_0272989X221104094 – Supplemental material for Opportunities and Challenges When Using the Electronic Health Record for Practice-Integrated Patient-Facing Interventions: The e-Assist Colon Health Randomized Trial [file sj-docx-1-mdm-10.1177_0272989X221104094.docx]

**Appendix Table A-1. Comparison of Those Who Completed/Did Not Complete the Baseline Survey among the Intent-to-Treat Sample (N=1,825)**

|  | **Completed Baseline Survey (N=1,419)** | **Did Not Complete Baseline Survey**  **(n=406)** | **P-value** |
| --- | --- | --- | --- |
| ***Continuous, mean (SD)*** |  |  |  |
| Age | 59.6 (7.2) | 59.9 (7.4) | 0.43 |
| Charlson Comorbidity Score | 1.1 (1.8) | 1.2 (1.9) | 0.34 |
| ***Discrete, n (%)*** |  |  |  |
| Race (missing=97) |  |  | 0.19 |
| White | 861 (63.8%) | 224 (59.3%) |  |
| Black | 399 (29.6%) | 121 (32.0%) |  |
| Other | 90 (6.7%) | 33 (8.7%) |  |
| Gender |  |  | 0.03 |
| Female | 906 (63.8%) | 234 (57.6%) |  |
| Male | 513 (36.2%) | 172 (42.4%) |  |
| Insurance (missing=1) |  |  | 0.08 |
| Commercial | 927 (65.5%) | 270 (66.5%) |  |
| Medicaid | 36 (2.5%) | 10 (2.5%) |  |
| Medicare | 436 (30.7%) | 426 (31.0%) |  |
| Other | 19 (1.3%) | 0 (0%) |  |
| Marital Status (missing=8) |  |  | 0.11 |
| Married | 916 (64.8%) | 279 (69.2%) |  |
| Single | 498 (35.2%) | 124 (30.8%) |  |
| Preferred Language (missing=51) |  |  | 0.72 |
| English | 20 (1.4%) | 4 (1.0%) |  |
| Other Language | 1369 (98.6%) | 381 (99.0%) |  |
| CRC Screening Order |  |  | 0.80 |
| Colonoscopy | 1146 (80.8%) | 325 (80.0%) |  |
| Stool test only | 273 (19.2%) | 81 (20.0%) |  |
|  |  |  |  |

CRC: colorectal cancer
